# Intensified Induction Chemotherapy in Locally Advanced Squamous Cell Carcinoma of the Anus—A Population-Based Experience from the Danish Anal Cancer Group

**DOI:** 10.3390/cancers13133226

**Published:** 2021-06-28

**Authors:** Karen Lycke Wind, Lisbeth Riber, Birgitte Mayland Havelund, Eva Serup-Hansen, Camilla Kronborg, Mette Marie Fode, Anders Jakobsen, Karen-Lise Garm Spindler

**Affiliations:** 1 Department of Experimental Clinical Oncology, Aarhus University Hospital, 8200 Aarhus, Denmark; k.g.spindler@rm.dk; 2 Department of Oncology, Vejle Hospital, University Hospital of Southern Denmark, 7100 Vejle, Denmark; Lisbeth.Riber@rsyd.dk (L.R.); Birgitte.Mayland.Havelund@rsyd.dk (B.M.H.); Anders.Jakobsen@rsyd.dk (A.J.); 3 Department of Oncology, Herlev and Gentofte Hospital, 2730 Herlev, Denmark; Eva.Serup-Hansen@regionh.dk; 4 Danish Centre for Particle Therapy, Aarhus University Hospital, 8200 Aarhus, Denmark; cam.kro@auh.rm.dk; 5 Department of Oncology, Aarhus University Hospital, 8200 Aarhus, Denmark; mettfode@rm.dk

**Keywords:** anus neoplasms, squamous cell carcinoma, induction chemotherapy, radiotherapy, chemoradiotherapy

## Abstract

**Simple Summary:**

The primary treatment modality for anal cancer is chemoradiotherapy, but patients with locally advanced disease (i.e., large tumors and/or involvement of regional lymph nodes) have a high risk of treatment failure. The use of chemotherapy prior to radiotherapy (induction chemotherapy) can potentially shrink the tumor and/or eradicate small cancer cells with metastatic potential, with a chance of a better outcome. With this paper, the authors present 20 years of nationwide experience with intensified induction chemotherapy in the treatment of locally advanced anal cancer, which indicates a role for further investigation in the most advanced cases.

**Abstract:**

Locally advanced squamous cell carcinoma of the anus (LASCCA) has a poor prognosis with a high risk of treatment failure calling for intensified therapy. We present the long-term follow-up of a nationwide cohort of LASCCA treated with intensified induction chemotherapy (ICT). The study included patients with LASCCA (T3-4N0 or T1-4N+) treated with at least one cycle of ICT (cisplatin, ifosfamide, leucoverin, and 5-flourouracil) between 1998–2018. Data were retrospectively collected from medical records, and statistics were performed in STATA 16.1. In total, 166 patients with LASCCA were identified. Following ICT, 157 patients (95%) received primary curative treatment with either radiotherapy (70%), chemoradiotherapy (27%), or abdominal perineal resection (3%). The overall local tumor response rate after ICT was 76% with 20 (13%) achieving complete local tumor response. After the primary treatment, 123 patients (79%) obtained complete response, and 27 underwent salvage surgery due to persistent disease. The median follow-up time was 6 years, local and distant failure rates 22% and 13%, respectively. The 3- and 5-year disease-free survival rates were 70% and 67%, and the 3- and 5-year overall survival rates were 76% and 70%, respectively. Intensified ICT regimen could be a supplementary treatment option in the most advanced cases of LASCCA. Prospective randomized trials are needed to investigate this approach further.

## 1. Introduction

Squamous cell carcinoma of the anus (SCCA) is a rare malignancy with an incidence between 0.5–1.7 per 100,000 per year in western countries [1]. The standard treatment is chemoradiotherapy (CRT), which ensures local control for the majority of patients [2]. However, locally advanced SCCA (LASCCA) (tumors ≥5 cm and/or regional lymph node metastases) has a poor outcome with high local and distant failure rates [3,4,5,6]. Radiotherapy treatment planning in patients with large tumors and multiple lymph node involvement can be complicated by complex target delineation and large target volumes resulting in unacceptable toxicity, and in some advanced cases definitive radiotherapy is not possible. Therefore, these high-risk patients hold a therapeutic challenge, and new treatment strategies to improve the outcome are urgently needed. In this context, induction chemotherapy (ICT) could be a method to downsize before definitive CRT and/or to eradicate micrometastases with the perspective of a better prognosis.

Two randomized trials [7,8] investigating ICT in SCCA have been published, but were not able to show a superior benefit from ICT. However, these trials investigated multiple treatment comparison in all TNM stages and were consequently not statistically designed to determine the effect of ICT in the most advanced stages. A limited number of studies have directly investigated ICT, but with different inclusion criteria, methodological approaches, and small sample sizes. The most commonly used ICT regimen in SCCA has been cisplatin and 5-flourouracil. Encouraging response rates after regimens consisting of ifosfamide and 5-flourouracil in combination with cisplatin and/or leucovorin have been reported in advanced and recurrent cervical cancer [9,10,11,12].

With this nationwide population-based study, we present 20 years of experience and long-time follow-up after intensified ICT consisting of cisplatin, ifosfamide, leucovorin, and 5-flourouracil administered prior to definitively intended treatment in patients with LASCCA.

## 2. Materials and Methods

### 2.1. Patients

Patients with T3-T4N0 or T1-4N+ treated with at least one cycle of ICT consisting of cisplatin, ifosfamide, leucovorin, and 5-fluorouracil between 1998 and 2018 were identified through internal registers at the three National Centers treating SCCA: Aarhus University Hospital, Vejle Hospital, and Herlev Hospital. Patient and tumor characteristics, treatment information, treatment response, and outcome data were collected retrospectively from medical records. Collection of data were approved by the Danish Patient Safety Authority (3-3013-2447/1) and the Danish Data Protection Agency (1-16-02-66-18).

The standard diagnostic work-up contained a clinical examination including digital anal examination and endoscopy in combination with either chest X-ray and computer tomography (CT) of the abdomen and pelvis or CT of the thorax, abdomen, and pelvis. In some cases, the diagnostic work-up also consisted of magnetic resonance imaging (MRI) of the pelvis and transrectal ultrasound, and 18-fluorodeoxyglucose positron emission tomography (FDG PET) CT was introduced as a diagnostic imaging modality during the study period.

Tumor staging is presented by the TNM-classification according to the American Joint Commission on Cancer (AJCC) staging system edition 7 [13] and 8 [14].

### 2.2. Treatment

Induction chemotherapy was administered over 2 days every 4 weeks. The ICT regimen included cisplatin (37.5 mg/m^2^ intravenously (iv.), ifosfamide (2.0 mg/m^2^ iv.), and 5-flourouracil (500 mg/m^2^ iv.) day one and two together with leucovorin (60 mg/m^2^ iv.) and mesna (500 mg/m^2^ iv.) administered before ifosfamide and 1g/m^2^ orally at 2 and 6 hours after ifosfamide). Granulocyte colony-stimulating factor (G-CSF) was not standard, but was used at the investigator’s discretion and further supportive care as per local practice.

Prescribed curative radiotherapy doses to tumor and pathological lymph nodes were 50–64 Gray (Gy) in 25–32 fractions and 46–54 Gy in 25–32 fractions to the elective nodal areas in five fractions per week. Gross tumor volumes (GTV) were defined based on post-ICT restaging with clinical and radiographic information. Standard elective nodal areas included perirectal, presacral, internal iliac, external iliac, inguinal lymph node areas and the mesorectum depending on the location of the primary tumor and boost to pathological lymph nodes. Clinical target volume margins differed during the 20-year timespan but were generally between 10–20 mm. Treatment was delivered in supine position with 3D conformal technique (1998–2007) or intensity modulated radiation technique (IMRT) (2007–2018). Therapeutic CT was used in all cases with or without MRI and FDG PET-CT in the treatment planning. Concomitant chemotherapy consisted of two cycles of cisplatin and 5-flourouracil, monotherapy with weekly cisplatin, or oral fluoropyrimidine.

### 2.3. Response Evaluation, Treatment Failure and Follow-Up

A clinical response evaluation of the tumor and clinical accessible lymph nodes was performed to assess the efficacy of ICT and to evaluate if curative treatment was possible. Six to eight weeks post radiotherapy a clinical evaluation was performed to assess the efficacy of the primary curative treatment (ICT + definitive radiotherapy/CRT/APR). The standard follow-up program consisted of a clinical examination every 3 to 4 months the first 2 years, then every 6 months for 2 years and after this, every year until 5 years. Imaging during follow-up was used when appropriate.

Complete local tumor response after ICT was defined as the absence of tumor and pathological lymph nodes evaluated by clinical examination. Complete response (CR) after radiotherapy was defined as absence of disease within 6 months after completing radiotherapy evaluated by clinical examination, imaging, and biopsy as appropriate. Persistent disease was defined as residual disease within 6 months after completion of radiotherapy. Treatment failure within the irradiated area after initial complete response was classified as locoregional recurrence. Distant recurrence was defined as metastasis outside the irradiated area after the initial complete response at any point during follow-up.

### 2.4. Statistical Methods

The statistical analyses were performed using STATA 16.1 (STATA/IC16.1, Stata Corp LP, College Station, TX, USA), and survival curves were estimated using the Kaplan–Meier method.

Recurrence was calculated from the date of diagnosis to the date of recurrence (locoregional and/or distant). Locoregional recurrence rate was calculated using the first event of locoregional recurrence. Distant failure rate was calculated using any first distant failure independent of earlier locoregional recurrence.

Overall survival (OS) (*n* = 166) was calculated from the time of diagnosis to death from any cause or date of last observation. Disease-free survival (DFS) on the intended to treat population (*n* = 166) was calculated from the date of diagnosis to progression, recurrence (locoregional or distant), death, or date of last observation—whichever came first. Patients who underwent salvage surgery due to persistent disease were defined as having completed combined curative treatment (ICT + definitive CRT/radiotherapy/APR + salvage surgery in case of persistent disease) and thereby achieved complete response. In the subgroup of patients completing combined curative treatment (*n* = 151), recurrence-free survival (RFS) was calculated from the date of diagnosis to the date of first recurrence (locoregional or distant), death, or date of last observation—whichever came first.

## 3. Results

### 3.1. Pre-Treatment Cohort Characteristics

A total of 166 patients with LASCCA who received at least one cycle of ICT between 1998 and 2018 were identified at the three Hospitals: Aarhus University Hospital (*n* = 117), Vejle Hospital, University Hospital of Southern Denmark (*n* = 42), and Herlev and Gentofte Hospital (*n* = 7). Pre-treatment characteristics are shown in Table 1. The mean age was 58 (range 29–79) years; the majority of patients were female (79%) and in good WHO performance status (PS0-1, 82%). Most tumors were classified as T3 or T4 tumors (82%) including 38% infiltrating nearby organs (T4 tumors) and 67% with regional lymph node metastases.

### 3.2. Therapy

Treatment characteristics are summarized in Table 2. The majority of patients received the intended three cycles (*n* = 130 (78%), range 1–5) of ICT.

A summary of adverse events during ICT is shown in Table 3. In brief, one potential ICT related death was recorded (0.6%), and 42 patients (25%) were hospitalized due to treatment related toxicity. The most common reason for treatment related hospitalization was neutropenic fever (71%) followed by nausea, vomiting, and/or dehydration (10%).

**Figure 1:**
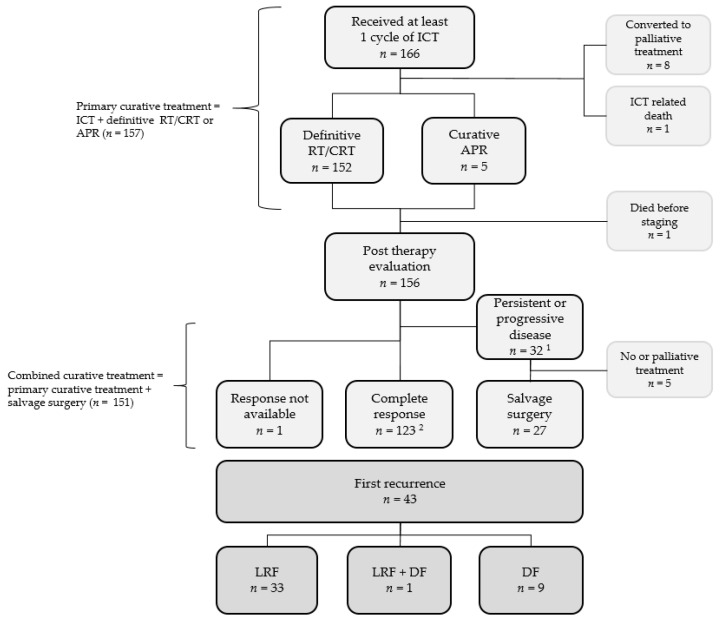
Flowchart of completed therapy, treatment response, and recurrence. ^1^ Persistent disease (*n* = 28) and progressive disease (*n* = 4). ^2^ Including three patients with no tumor cells in the specimen after salvage surgery. Abbreviations: RT, radiotherapy; CRT, chemoradiotherapy; APR, abdominal perineal resection; LRF, locoregional failure; DF, distant failure.

A summary of the completed therapy is shown in Figure 1. Definitive radiotherapy was prescribed for 152 cases (CRT *n* = 42, radiotherapy only *n* = 110). No major radiotherapy delays were observed, but in five cases, radiotherapy was omitted and patients underwent abdominal perineal resection (APR) after ICT. In further eight cases, the planned curative treatment was not possible (Figure 1).

### 3.3. Treatment Response

The response rates after ICT are summarized in Table 4. Complete local tumor response was achieved by 20 patients (13%), and an additional 98 (63%) achieved a partial response (PR). The overall local tumor response rate after ICT alone was 75%.

Within 6 months after primary curative treatment, 123 patients (79%) achieved CR, whereas 32 (21%) had persistent disease. Altogether, 91% successfully completed the combined curative treatment. Not including salvage surgeries for persistent disease, 74% completed primary curative treatment.

The median follow-up time was 6 years (range 0.3–21.5), and 92 patients (55%) were still alive at the time of the last follow-up. Of the 156 patients who completed the combined curative treatment, 43 patients developed recurrence either locoregional (*n* = 34) and/or distant (*n* = 20) with an overall locoregional recurrence rate of 22% and a distant failure rate of 13%. Successful salvage surgery was performed in nine cases. Of note, in patients with tumors infiltrating others organs a good local control was observed with a locoregional recurrence rate of 12%.

### 3.4. Survival Analysis

**Figure 2:**
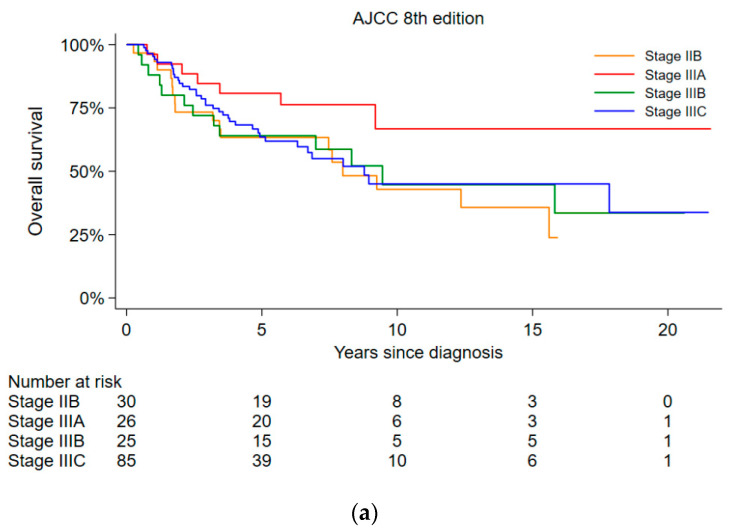

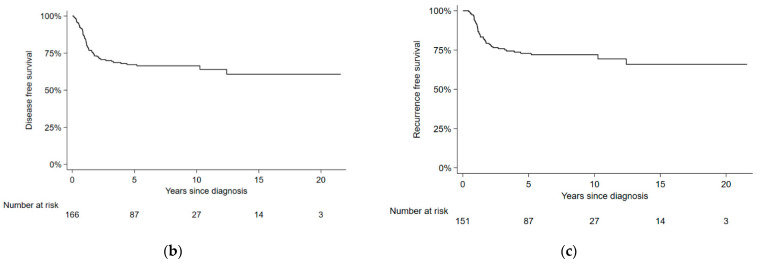
Kaplan–Maier survival curves. (**a**) Overall survival by stages (*n* = 166); (**b**) disease-free survival (*n* = 166); (**c**) recurrence-free survival in patients completing combined curative treatment (*n* = 151).

Kaplan–Meier survival plots are depicted in Figure 2. Overall survival by stages is presented in Figure 2a. For all stages together, the 3- and 5-year OS was 76% and 67%. The 3- and 5-year DFS in the intended to treat population (*n* = 166) was 70% and 67%, respectively (Figure 2b). The 3- and 5-year RFS was 76% and 73% (Figure 2c), in the group of patients who successfully completed the combined curative modality course (*n* = 151).

## 4. Discussion

We present the outcome of a large nationwide cohort of patients within the most advanced group of LASCCA. In the literature, the 3-year DFS in patients with tumors ≥ 5 cm ± lymph node positive disease treated with CRT alone is reported between 30% and 65% [3,4,15], indicating a need for an intensified treatment strategy for these high-risk patients. The results presented here indicates a considerable effect of ICT in LASCCA with three of four patients having partial or complete response and 91% completing combined curative treatment. Of these, 74% achieved CR without salvage surgery.

The current literature on the outcome after ICT in LASCCA is characterized by a small number of heterogeneous studies, and any conclusion from comparison with the present results is difficult and should be drawn with caution. However, ICT has been investigated in two randomized trials—the RTOG-9811 study [7] and the ACCORD 03 trial [8]. These trials did not demonstrate a significant benefit from ICT, but in both studies, the designs included multiple treatment comparison and, more importantly, patients with early-stage disease. In the RTOG-9811 trial, 65% patients were stage I-II compared to only 18% in our study. Only 27% of tumors were larger than 5 cm compared to 72% in our cohort. Finally, 67% of our patients presented with regional lymph node metastases compared to only 30% in the RTOG-9811 trial. In brief, the RTOG-9811 trial included patients with a good prognosis a priori, and it can be anticipated that the majority of these patients would not benefit from systemic treatment due to early localized and small volume disease. Similarly, in the ACCORD 03 trial only 23% of patients had N2 or N3 disease compared to 50% in our study. In addition, 37% were early stage T1-T2N0-N1 compared to only 3% in our study. Consequently, these trials are not comparable to our cohort and cannot be concluded to disqualify ICT as an option in the most advanced cases.

A few small studies have reported the use of ICT in LASCCA and a 3-year DFS of approximately 67% [16,17] in line with the 3-year DFS in our study. In a population-based study from Sweden [18] a subgroup of 91 patients with LASCCA (tumors more than 4 cm or lymph node positive) were treated with ICT consisting of cisplatin or carboplatin in combination with 5-flourouracil. Patients who received ICT had a significantly better 5-year OS of 63%, compared to patients with LASCCA not receiving ICT with a 5-year OS of 44%. This supports further investigation of ICT, in the most advanced cases.

In our cohort of the most advanced tumors, treated with an intensified ICT regimen, we observed a promising overall response rate (CR and PR) after ICT of 75% with 13% achieving CR and 63% PR. The ICT did not seem to compromise the full treatment course with only minor treatment delays and a high fraction completing the combined treatment course. No other data have been reported using this triple combination. In retrospective comparison, the overall response rate after ICT with doublet cisplatin and 5-flourouracil varied between 61–71% and the CR and PR rates between 10–18% and 51–60%, respectively [8,16,17]. In the metastatic setting only moderate response rates have been reported after doublet cisplatin and 5-flourouracil [19,20]. Furthermore, CR rates in the metastatic setting with cisplatin, 5-flourouracil, and docetaxel were 42% in the Epitopes-HPV02 study [21] compared to 5.6% with doublet cisplatin and 5-flourouracil as reported in a small French study [22]. Conclusively, this supports the investigation of a more intensified regimen.

The possible advantage of intensified therapy should be carefully balanced against the risk of treatment complications and used for selected cases. In the present study, the majority of grade 3–4 treatment related toxicity was neutropenic fever, and we observed one potential ICT related death (0.6%). By contrast, while the toxicity profile may vary, the overall frequency of grade 5 events was 2% in a combined Nordic report on patients treated with various chemotherapy or CRT regimens [23]. We observed a relatively high rate of treatment-related hospitalization of 23% during ICT. However, with doublet cisplatin and 5-flourouracil alone the rate of grade 3–4 toxicity has been reported between 15 and 33% [8,17]. While a more intensified treatment strategy for high-risk SCCA is urgently needed, there is an unmet need to define effective treatment options for ICT with feasible toxicity profiles. Prospective recording of both toxicity and quality of life data following intensified regimens will be mandatory for further development. Bone marrow support with G-CSF was not routinely used in our cohort. However, we did observe a relatively high rate of neutropenic fever, and it could be argued that the use of G-CSF in relation to this intensified regimen should be standard supportive care. According to the ESMO Clinical Practice Guideline [24], G-CSF is recommended for high risk of neutropenic fever (>20%). The use of prophylactic G-CSF would probably lower the rate of neutropenic fever and consequent hospitalization during this intensified treatment.

In this study, we observed a locoregional recurrence rate of 22% and a distant recurrence rate of 13% for patients who completed combined curative treatment, and of the 156 patients who completed curative treatment 32 had persistent disease. This cohort is of the most advanced cases, and a high locoregional recurrence rate is expected; however, a distant failure rate of 13% among these high-risk patients is low. The use of ICT potentially lowers the risk of distant failure by sterilizing micrometastases, and add to downsizing of large tumors thereby decreasing volume and possibly acute and late morbidity. Further intensification of the local treatment (to lower the risk of local failures) should also be considered in future studies, by exploring new radiotherapy techniques and concomitant systemic options like immunotherapy [25,26]. Nevertheless, there is an urgent need for tools to improve the selection of patients who will benefit from ICT prior to CRT [27,28].

An aspect to consider is the overall treatment time (OTT). Induction chemotherapy prolongs OTT and this could be of concern to local control as shown in a pooled analysis of RTOG-8704 and -9811 [29], where the authors found that OTT was significantly associated to local failure, but not OS. However, the effect of OTT versus radiation treatment time (RTT) was investigated in locally advanced head and neck squamous cell carcinomas treated with ICT prior to radiotherapy, and here, the authors found that prolonged RTT was significantly associated with locoregional recurrence and OS, whereas prolonged OTT was not associated with these outcomes, when adjusted for RTT [30]. The concern of prolonged RTT is based on the risk of accelerated repopulation, and split course radiotherapy is no longer standard [31,32]. Overall treatment time should also be taken into consideration when treating patients with high risk of distant recurrence, but the direct risk from prolonged OTT due to ICT is not clear.

The data presented here have limitation due to its retrospective nature, but is to our knowledge the largest retrospective dataset on LASCCA treated with ICT, and due to the national registers, we were able to present a data set with very few missing data.

To investigate the use of intensified ICT in the most advanced cases of LASCCA, prospective randomized trials including only the most advanced cases are needed. Future trials should include a control arm of CRT only to compare the effect of ICT in this setting. Here, we describe a cohort treated according to historical practice.

## 5. Conclusions

In conclusion, this is the first study to report on an intensified ICT strategy for LASCCA. Even though ICT is not a recommended standard option, the high response rates, DFS, and OS show proof-of principle of using an intensified ICT regimen in the most advanced cases calling for further clinical investigation.

## Figures and Tables

**Table 1:** Pre-treatment characteristics.

Patient Characteristics	*n* = 166 (%)
Age (year)	58 (29–79)
Median (range)	
Gender	
Female	131 (79)
Male	35 (21)
WHO performance status	
0	99 (60)
1	3 (22)
≥2	4 (2)
Not available	27 (16)
Tumor size (cm)	-
Median (range) ^1^	6 (1–12)
T-stage (AJCC 7th [13] and 8th Edition [14])	-
Tx	5 (3)
T1	1 (1)
T2	24 (14)
T3	73 (44)
T4	63 (38)
N-stage (AJCC 8th Edition [14])	-
N0	55 (33)
N1a	88 (53)
N1b	3 (2)
N1c	20 (12)
N-stage (AJCC 7th Edition [13])	-
N0	55 (33)
N1	27 (17)
N2 ^2^	42 (25)
N3	42 (25)
Lymph node location ^3^	-
Perirectal	58 (52)
Inguinal, unilateral	49 (44)
Inguinal, bilateral	23 (21)
Internal iliac	17 (15)
External iliac	24 (22)
Stage (AJCC 8th edition [14])	-
Stage IIB	30 (18)
Stage IIIA	26 (16)
Stage IIIB	25 (16)
Stage IIIC	85 (51)
Stage (AJCC 7th edition [13])	-
Stage II	30 (18)
Stage IIIA	42 (25)
Stage IIIB	94 (57)
P16 status	-
Unknown p16 status	118 (71)
P16 positive	44 (27)
P16 negative	4 (2)

^1^ Clinical evaluation of largest tumor diameter. In case of missing clinical tumor size, the largest MRI tumor size was used. ^2^ Including external iliac lymph node metastasis. ^3^ Location of lymph nodes in patients with node positive disease (*n* = 111). Patients may have more than one location, percentage therefore a sum more than 100. Abbreviations: AJCC, American Joint Committee on Cancer.

**Table 2:** Treatment characteristics.

**Induction Chemotherapy**	***n* = 166 (%)**
Cycle of ICT	-
1 cycle	11 (7)
2 cycles	22 (13)
3 cycles	130 (78)
>3 cycles	3 (2)
**Definitive Radiotherapy**	***n* = 152 (%)**
Prescribed doses	-
64/51.2 Gy/32F	115 (76)
60.2/50.4 Gy/28F	22 (14)
60/49.5 Gy/30F	5 (3)
60/50 Gy/30F	3 (2)
Other	7 (5)
Technique	-
IMRT	108 (71)
3D-conformal radiotherapy	44 (29)
**Concomitant Chemotherapy**	***n* = 42 (%)**
Cisplatin and fluorouracil	4 (10)
Weekly cisplatin	21 (50)
Oral fluorouracil	17 (40)

Abbreviations: ICT, induction chemotherapy; Gy, Gray; IMRT, intensity modulated radiation technique.

**Table 3:** Adverse events related to induction chemotherapy.

**Dose Reduction or Discontinuation**	***n* = 166 (%)**
No dose reduction or discontinuation	99 (59)
Dose reduction or discontinuation of one agent	46 (28)
Discontinuation of one or more cycles	18 (11)
Not available	3 (2)
**Hospitalization**	***n* = 166 (%)**
No hospitalization during ICT	103 (62)
Hospitalization during ICT related to ICT	42 (25)
Hospitalization during ICT NOT related to ICT	12 (7)
Not available	9 (5)
**Hospitalization with Possible Relation to ICT**	***n* = 42 (%)**
Febrile neutropenia	30 (71)
Dehydration/nausea/vomiting	4 (10)
Encephalopathy	2 (5)
Anemia	2 (5)
Renal toxicity	1 (2)
Diarrhea	1 (2)
Fever without neutropenia	1 (2)
Unknown cause	1 (2)

**Table 4:** Response.

**Response After ICT ^1^**	***n* = 156 (%)**
Complete local tumor response	20 (13)
PR	98 (63)
SD	26 (16)
PD	12 (8)
**Post Therapy Evaluation ^1^**	***n* = 155 (%)**
CR	123 (79)
Persistent disease	28 (18)
PD	4 (3)

^1^ Response evaluation after ICT was not available in nine patients and in one patient post therapy, and these were excluded from response rate analysis. Abbreviations: CR, complete response; PR, partial response; SD, stable disease; PD, progression disease.

## Data Availability

Data not available due to legal restrictions.
